# Supplementary material for: Smooth Interpolating Curves with Local Control and Monotone Alternating Curvature
Source: Comput Graph Forum. 2022 Oct 6;41(5):25–38. doi: 10.1111/cgf.14600 (PMC9827861; doi:10.1111/cgf.14600)
Supplement: Supplementary file 1 — Supplement Material [file CGF-41-25-s001.zip › Local-Smooth-Interpolating-MonoCurvature/extern/clothoids/submodules/quarticRootsFlocke/docs/api-cpp/program_listing_file_PolynomialRoots-1-Quadratic.cc.html]

Program Listing for File PolynomialRoots-1-Quadratic.cc — Quartic Roots v1.0.0

### Navigation

- index
- toc
- Quartic Roots »
- Program Listing for File PolynomialRoots-1-Quadratic.cc

# Program Listing for File PolynomialRoots-1-Quadratic.cc¶

↰ Return to documentation for file (`PolynomialRoots-1-Quadratic.cc`)

```
/*--------------------------------------------------------------------------*\
 |                                                                          |
 |  Copyright (C) 2017                                                      |
 |                                                                          |
 |         , __                 , __                                        |
 |        /|/  \               /|/  \                                       |
 |         | __/ _   ,_         | __/ _   ,_                                |
 |         |   \|/  /  |  |   | |   \|/  /  |  |   |                        |
 |         |(__/|__/   |_/ \_/|/|(__/|__/   |_/ \_/|/                       |
 |                           /|                   /|                        |
 |                           \|                   \|                        |
 |                                                                          |
 |      Enrico Bertolazzi                                                   |
 |      Dipartimento di Ingegneria Industriale                              |
 |      Universita` degli Studi di Trento                                   |
 |      email: enrico.bertolazzi@unitn.it                                   |
 |                                                                          |
\*--------------------------------------------------------------------------*/

#ifdef __GNUC__
#pragma GCC diagnostic ignored "-Wunused-function"
#endif
#ifdef __clang__
#pragma clang diagnostic ignored "-Wglobal-constructors"
#pragma clang diagnostic ignored "-Wvla-extension"
#pragma clang diagnostic ignored "-Wvla"
#pragma clang diagnostic ignored "-Wunused-function"
#pragma clang diagnostic ignored "-Wdocumentation-unknown-command"
#endif

#include "PolynomialRoots.hh"

#include <cmath>
#include <iostream>
#include <algorithm>
#include <limits>

#ifndef DOXYGEN_SHOULD_SKIP_THIS

namespace PolynomialRoots {

  using  std::abs;
  static valueType const machepsi = std::numeric_limits<valueType>::epsilon();

  indexType
  Quadratic::getRealRoots( valueType r[] ) const {
    indexType nr = 0;
    if ( !cplx ) {
      r[nr++] = r0;
      if ( nrts > 1 ) r[nr++] = r1;
    }
    return nr;
  }

  indexType
  Quadratic::getPositiveRoots( valueType r[] ) const {
    indexType nr = 0;
    if ( !cplx ) {
      if ( nrts > 0 && r0 > 0 ) r[nr++] = r0;
      if ( nrts > 1 && r1 > 0 ) r[nr++] = r1;
    }
    return nr;
  }

  indexType
  Quadratic::getNegativeRoots( valueType r[] ) const {
    indexType nr = 0;
    if ( !cplx ) {
      if ( nrts > 0 && r0 < 0 ) r[nr++] = r0;
      if ( nrts > 1 && r1 < 0 ) r[nr++] = r1;
    }
    return nr;
  }

  indexType
  Quadratic::getRootsInRange( valueType a, valueType b, valueType r[] ) const {
    indexType nr = 0;
    if ( !cplx ) {
      if ( nrts > 0 && r0 >= a && r0 <= b ) r[nr++] = r0;
      if ( nrts > 1 && r1 >= a && r1 <= b ) r[nr++] = r1;
    }
    return nr;
  }

  indexType
  Quadratic::getRootsInOpenRange( valueType a, valueType b, valueType r[] ) const {
    indexType nr = 0;
    if ( !cplx ) {
      if ( nrts > 0 && r0 > a && r0 < b ) r[nr++] = r0;
      if ( nrts > 1 && r1 > a && r1 < b ) r[nr++] = r1;
    }
    return nr;
  }

  void
  Quadratic::eval( valueType x, valueType & p, valueType & dp ) const {
    valueType const & A = ABC[0];
    valueType const & B = ABC[1];
    valueType const & C = ABC[2];
    if ( std::abs(x) > 1 ) {
      valueType z  = 1/x;
      valueType x2 = x*x;
      p = ((C*z+B)*z+A)*x2;
    } else {
      p = (A*x+B)*x+C;
    }
    dp = 2*A*x+B;
  }

  /*\
   *  Calculate the zeros of the quadratic a*z^2 + b*z + c.
   *  The quadratic formula, modified to avoid overflow, is used
   *  to find the larger zero if the zeros are real and both
   *  are complex. The smaller real zero is found directly from
   *  the product of the zeros c/a.
  \*/

  void
  Quadratic::findRoots() {
    valueType const & A = ABC[0];
    valueType const & B = ABC[1];
    valueType const & C = ABC[2];

    r0 = r1 = 0;
    nrts = 0;
    cplx = dblx = false;

    if ( isZero(A) ) { // less than two roots b*z + c = 0
      if ( !isZero(B) ) { nrts = 1; r0 = -C/B; }
    } else if ( isZero(C) ) { // a*z^2 + b*z  = 0
      nrts = 2;
      dblx = isZero(B);
      if ( !dblx ) {
        r0 = -B/A;
        if ( r0 < 0 ) std::swap(r0,r1);
      }
    } else { // Compute discriminant avoiding overflow.
      valueType hb    = B/2; // b now b/2
      valueType abs_b = std::abs(hb);
      valueType abs_c = std::abs(C);
      valueType e, d;
      if ( abs_b < abs_c ) {
        e = C < 0 ? -A : A;
        e = (hb*hb)-e*abs_c;
        d = std::sqrt(std::abs(e));
      } else {
        e = 1 - (A/hb)*(C/hb);
        d = std::sqrt(std::abs(e))*abs_b;
      }
      nrts = 2;
      cplx = e < 0;
      if ( cplx ) {
        // complex conjugate zeros
        r0 = -hb/A;         // real part
        r1 = std::abs(d/A); // immaginary part
      } else {
        // real zeros
        dblx = isZero(d);
        if ( dblx ) {
          r0 = r1 = -hb/A;
        } else {
          if ( hb >= 0 ) d = -d;
          r0 = (d-hb)/A;
          //r1 = (-d-hb)/a;
          if ( !isZero(r0) ) r1 = (C/r0)/A;
          if ( r0 > r1 ) std::swap(r0,r1); // order roots
        }
      }
    }
  }

  void
  Quadratic::info( std::ostream & s ) const {
    valueType const & A = ABC[0];
    valueType const & B = ABC[1];
    valueType const & C = ABC[2];
    s << "\npoly A=" << A << " B=" << B << " C=" << C
      << "\nn. roots = " << nrts
      << "\ncomplex  = " << (cplx?"YES":"NO")
      << "\ndouble   = " << (dblx?"YES":"NO");
    if ( cplx ) {
      s << "\nx0 = (" << r0 << "," <<  r1 << ')'
        << "\nx1 = (" << r0 << "," << -r1 << ')';
    } else if ( dblx ) {
      s << "\nx0 = x1 = " << r0;
    } else if ( nrts == 1 ) {
      s << "\nx0 = " << r0;
    } else if ( nrts == 2 ) {
      s << "\nx0 = " << r0
        << "\nx1 = " << r1;
    }
    s << '\n';
  }

  bool
  Quadratic::check( std::ostream & s ) const {
    valueType const & A = ABC[0];
    valueType const & B = ABC[1];
    valueType const & C = ABC[2];
    bool ok = true;
    valueType epsi = 10 * ( std::abs(A) +
                            std::abs(B) +
                            std::abs(C) ) * machepsi;
    if ( cplx ) {
      valueType z0 = std::abs(eval( root0() ));
      valueType z1 = std::abs(eval( root1() ));
      s << "|p(r0)| = " << z0
        << "\n|p(r1)| = " << z1
        << '\n';
      ok = z0 < epsi && z1 < epsi;
    } else if ( nrts == 1 ) {
      valueType z0 = eval( real_root0() );
      s << "p(r0) = " << z0  << '\n';
      ok = std::abs(z0) < epsi;
    } else if ( nrts == 2 ) {
      valueType z0 = eval( real_root0() );
      valueType z1 = eval( real_root1() );
      s << "p(r0) = " << z0
        << "\np(r1) = " << z1
        << '\n';
      ok = std::abs(z0) < epsi && std::abs(z1) < epsi;
    }
    return ok;
  }

}

#endif

// EOF: PolynomialRoots-1-Quadratic.cc
```

### Quick search

### Table of Contents

- Quartic Roots Solver
- C++ API

«
hide menu

menu
sidebar
»

### Navigation

- index
- toc
- Quartic Roots »
- Program Listing for File PolynomialRoots-1-Quadratic.cc

© Copyright 2021, Enrico Bertolazzi.
Created using Sphinx 4.2.0.
